# Perception of venipuncture pain in children suffering from chronic diseases

**DOI:** 10.1186/1756-0500-7-735

**Published:** 2014-10-18

**Authors:** Sofia Bisogni, Chiara Dini, Nicole Olivini, Daniele Ciofi, Francesca Giusti, Simona Caprilli, José Rafael Gonzalez Lopez, Filippo Festini

**Affiliations:** PhD School in Nursing Science, University of L’Aquila, L’Aquila, Italy; Undergraduate BSN Course, University of Florence, Florence, Italy; Department of Health Sciences, University of Florence, Viale Pieraccini 24, 50139 Florence, Italy; Nursing Research Unit, Meyer Children Hospital of Florence, Viale Pieraccini 24, 50139 Florence, Italy; Psychology Unit, Meyer Children Hospital of Florence, Florence, Italy; Faculty of Nursing, University of Sevilla, Sevilla, Spain

## Abstract

**Background:**

Venipuncture pain in children results from a variety of co-factors which increase the intensity of the nociceptive stimulus. Among them, anticipatory anxiety plays an important role. Children with chronic diseases undergo invasive procedures and venipuncture more often than other children. Some healthcare professionals still believe that children who are repeatedly exposed to painful procedures, such as children with chronic diseases, gradually increase their pain tolerance and that, as a result, they have a higher pain threshold than children with no chronic diseases. The purpose of this study was to assess whether a difference exists in the perception of venipuncture pain between children with chronic diseases and children with no previous health problems nor experience of venipuncture.

**Methods:**

A cross-sectional study was carried out using the Wong and numeric pain scales and the Observational Scale of Behavioral Distress (OSBD) for the assessment of behavioral distress. A group of children with chronic diseases and a group of children with no previous health problems nor experience of venipuncture, aged 4 to 12 years, both boys and girls, were observed during a standardized venipuncture procedure.

**Results:**

The study included 230 children in total: 82 of them suffered from chronic diseases and had already experienced venipuncture at least once, while the remaining 148 children had no previous experience of venipuncture. The children with chronic diseases reported more pain (median pain score of 8 on the Wong or numeric scales,) and showed more signs of behavioral distress (median score of 27 on the OSBD) than non-chronic children (median pain score of 2 on the Wong/numeric scales, p = 0.00001; median OSBD score 5, p = 0.00001).

**Conclusions:**

Our study suggests that children with chronic diseases have a lower pain threshold than children of the same sex and age who experience venipuncture for the first time.

## Background

It is well established that children remember painful experiences [1, 2] and that the way they commit such experiences to memory conditions how they react to subsequent exposures to painful procedures [3, 4]. Indeed, what a child remembers about previous painful events is a good predictor of its response to future pains [3–6].

Recalling pain is a complex cognitive process involving different capabilities, such as encoding, storing and retrieving from memory the experienced pain. Grading recalled pain requires comparing retrieved pain with the pain rating scale. Such a complex cognitive process is likely influenced by the cognitive development of the child and the attention paid to the painful experience [7]. Some studies tried to assess whether pain memories in children are reliable and over what time period. A body of evidence shows that stressful procedures are recalled quite accurately over delays of between 6 weeks and many years [3, 7]. The moderate-high accuracy of children’s recall of pain intensities emerging from modern studies is in contrast to the findings of Lehmann et al. [8] and Lander et al. [9], who reported a lower accuracy. These divergence can be due to the use of pain intensity measures of different reliability and validity, suggesting that the main factor in children’s capability to correctly recall pain intensities is the way of measuring these intensities [7].

It is well established that memory reports of stressful experiences are influenced by many factors, that are similar to those affecting non-stressful experiences [3, 7, 10, 11]. In addition to situational and methodological influences -for example, the passage of time or the format of questioning- children’s reports of painful events can relate to individual differences, such as age, gender, temperament, anxiety, prior experiences and pain threshold [3]. Rocha et alii examined the effects of temperament and trait anxiety on memory of pain: trait-anxious children tended to recall more pain than they initially reported, thus indicating that their recollections of pain could have been distorted. Temperament, although it is believed to influence sensitivity and reactivity to stressful events, [12–15] did not show any significant effect on pain recollection [4]. Also, parental variables and strategies for coping pain carried out when performing a medical procedure can shape children’s memory of a stressful event, as the insertion of an intravenous needle [16].

As a matter of fact, most children view venipuncture as one of the most fearful aspects of attending hospital, be it for a scheduled medical examination or for an unexpected admission [17]. Even if some children are able to tolerate the procedure, most children frequently display high levels of pain, anxiety and fear [17, 18]. Pain upon venipuncture, considered as a specific aspect of what we call procedural pain, results from the interaction of several factors that are involved in the modulation of the nociceptive stimulus, among which anticipatory anxiety plays a major role [16, 17, 19–21]. A number of pharmacological and cognitive-behavioral interventions exist that are effective in treating and preventing pain and anticipatory anxiety [16, 17, 22–25].

Insufficient evidence exist specifically in regard to children affected by a chronic condition and their memory of previous painful experiences and it is not clear whether having a chronic condition affects a child’s memory of pain and how the factors influencing recalled pain also impact on the way chronic patients remind experienced pain and cope with new stressful stimuli [13, 26–28]. Some studies suggest that procedural pain is often undertreated in children with a chronic disease [29, 30]. A frequent belief among healthcare professionals is that children who are repeatedly exposed to painful procedures -such as those with a chronic condition- gradually increase their pain tolerance and that the more often they have undergone painful procedures, the more their perception of pain decreases [31–34]. The results of a recent study by Rømsing et al. [35] seem to confirm this conviction, showing that children who had experienced a painful procedure before reported significantly lower pain scores.

In order to avoid the risk that healthcare professionals pay insufficient attention to procedural pain in children with a chronic disease, it is necessary to verify the impact which minor invasive procedures, such as peripheral venous needle insertion, have in clinical practice on children with chronic diseases who have already undergone this procedure before. In particular, it is important to assess whether their perception of pain is different from the one of children who have never had any health problem before and who have no previous experience of venipuncture.

The purpose of this research was to compare the pain perceived and the behavioral distress shown during a standardized venipuncture by children with chronic diseases who have already been exposed to venipuncture, with those of a group of children who have never had any health problem before and who have no previous experience of venipuncture, in order to evaluate if differences exist.

## Methods

We carried out a cross-sectional study on a population of patients admitted to the Day Hospital service (DH) of a third level Italian children’s hospital.

We included Italian mother tongue children aged 4 to 12 years, both boys and girls, who came to the DH for a scheduled blood test.

In order to avoid the confounding effect that may be determined by the different types of pharmacological and cognitive-behavioral methods of pain control, we included in the study only the children whose parents decided to refuse pain control interventions. This was possible because, as previously reported by literature [35] a considerable part of parents do not want that interventions to reduce pain are used for venipuncture on their children, possibly due to cultural or religious reasons. Also, children who were under the effect of any drug for pain treatment were excluded from the study.

Parents’ informed consent was collected by one of the researchers.

The recruited children were divided into two groups. The first group was made up of children who were having their blood taken for the first time in their lives, who had never had any health problem before and who had never had to undergo any diagnostic test or treatment before. The second group was made up of children who, according to the definition of the National Center for Health Statistics [36], were suffering from a chronic disease, who had been monitored for at least two years by the endocrinology, hepatology, gastroenterology or immunology Units of the hospital and who had already experienced venipuncture and other invasive procedures before.

We used accidental, non-probability, convenience sampling. The participation to the study was proposed to the parents of all the children who accessed the DH consecutively during a period of two months. Right before the venipuncture, information regarding the procedure they were about to undergo was given to all the children by the Nurse in charge of the procedure, according to their age and cognitive developmental stage, as suggested by Duff et al. [37]. At least one of the parents participated to the procedure. All the parents were suggested to sit in front of the venipuncturing Nurse positioning their child on their lap as suggested by Wong [38]. When restraining the child was indispensable, it was performed by a parent using one of the therapeutic hugging methods suggest by Wong [38].

Each subject underwent a standardized venipuncture procedure, with the same needle type and gauge (23 gauge) being used. The venipunctures were performed by one of a group of three very experienced nurses. For every child who underwent venipuncture we recorded their sex and age.

Perceived pain was measured using a self-assessment scale: the Wong faces rating scale for children up to 7 years [39] and a numerical rating scale for older children, both with a score range from 0 to 10.

Behavioral distress was measured using the Observation Scale of Behavioral Distress (OSBD) [40], in which scores range from 0 to 33.

The pain self assessment scale was administered to the child by the venipuncuring Nurse, who recorded the score given by each child. The OSBD scale was scored by two researchers who were present during the venipuncture, who assessed independently the behavioral distress shown by the child during the performance of venipuncture, not communicating nor interacting with each other and with all the presents. Pain and behavioral distress were assessed only for the first attempt of venipuncture made by the nurses, irrespective of its success.

Finally, we analyzed the differences between the median pain and OSBD scores of chronic and of non-chronic children using the Wilcoxon-Mann–Whitney test.

The study was performed in accordance with the Declaration of Helsinki and was approved by the Ethics Committee of the Meyer Children University Hospital of Florence (Decision 279, February 1, 2010).

## Results

We recruited 230 children, 82 of whom suffered from chronic diseases and 148 of whom had no previous health problems nor experience of venipuncture. The overall mean age was 92 months. Boys accounted for 46% of the study population. Table 1 shows that, with respect to age and sex, there were no statistically significant differences between the group of chronic children and the group of children with no previous experience of venipuncture.

**Table 1:** **Mean age in months and percentage of males in the two groups of study**

	Chronic	Non-chronic	P
**Mean age (months)**	95.3	90.6	ns
**Males (%)**	41.5	48.6	ns

Table 2 shows the differences between the median self-reported pain and OSBD scores of the group of chronic children and of the group of children with no previous experience of venipuncture.

**Table 2:** **Median self-reported pain and OSBD scores of the two groups of study**

	Chronic	Non-chronic	Mann–Whitney-Wilcoxon test p
**Median OSBD score**	27 (range 18–33)	5 (range 0–33)	<0.00001
**Median pain score**	8 (range 6–8)	2 (range 0–10)	<0.00001

## Discussion and conclusions

Our study suggests that the children suffering from chronic diseases, whose clinical conditions cause them to be frequently exposed to invasive procedures and venipuncture, had significantly higher pain scores in both scales than the children with no previous experience of venipuncture. Chronic children thus seem to show more signs of pain and behavioral distress than children who have no chronic diseases. It is also important to note that, with respect to age, there are no statistically significant differences between the two groups of patients involved in the study. Several studies have indeed demonstrated that there is an inverse correlation between children’s age and their pain and distress levels during invasive procedures, as well as that pain tolerance increases with age [41].

This factor did not influence our results. The same holds true for gender [42], given that there are no statistically significant differences between boys and girls. Possible limitations of the study are the limited size of the study population and the use of non-random sampling.

Our study suggests that children suffering from chronic diseases tend to have a lower pain threshold than children of the same sex and age who have their blood taken for the first time.

This finding contradicts the common belief that children with chronic diseases get used to painful procedures over time and conflicts with the data presented by Romsing [35]. It also confirms the important role played by anticipatory anxiety in the perception of pain and in the development of behavioral distress associated with venipuncture [17, 21, 23].

Our study has some limitations. First, we did not assess in children some variables involved with the memory of previous pain -such as trait anxiety, coping style, temperament- that may have influenced our results. Secondly, we did not take into account the above variables in parents who accompanied their children during venipunctures.

The management of pain and of the negative reactions associated with it is a multidisciplinary concern: the collaboration of several professional figures can help reduce patient suffering. In a Pediatric Center that intends to provide high-quality care, paying attention to children’s experience of pain is of vital and primary importance.

For children in hospital, venipuncture is one of the most fearful and painful aspects, which makes them feel the most anxious. Therefore, in daily clinical practice it is necessary to increasingly promote the adoption of the effective and validated techniques known as systemic desensitization [17].

## References

[1] Cohen LL, Blount RL, Cohen RJ, Ball CM, McClellan CB, Bernard RS (2001). Children’s expectations and memories of acute distress: Short-and long-term efficacy of pain management interventions. J Pediatr Psychol.

[2] Ornstein PA, Manning EL, Pelphrey BA (1999). Children’s memory for pain. Dev Behav Pediatr.

[3] Von Baeyer CL, Marche TA, Rocha EM, Salmon K (2004). Children’s memory for pain: overview and implications for practice. J Pain.

[4] Rocha EM, Marche TA, von Baeyer CL (2009). Anxiety influences children’s memory for procedural pain. Pain Res Manage.

[5] Gedney JJ, Logan H (2006). Pain related recall predicts future pain report. Pain.

[6] Noel M, Chambers CT, McGrath PJ, Klein RM, Stewart SH (2012). The influence of children’s pain memories on subsequent pain experience. Pain.

[7] Zonnevelda LNL, McGrathb PJ, Reidd GJ, Sorbi MJ (1997). Accuracy of children’s pain memories. Pain.

[8] Lehmann HP, Bendebba M, DeAngelis C (1990). The consistency of young children’s assessment of remembered painful events. J Dev Behav Pediatr.

[9] Lander J, Hodgins M, Fowler-Kerry S (1992). Children’s pain predictions and memories. Behav Res Ther.

[10] Quas JA, Goodman GS, Bidrose S, Pipe M-E, Craw S, Ablin DS (1999). Emotion and memory: children’s long-term remembering, forgetting, and suggestibility. J Exp Child Psychol.

[11] Chen E, Zeltzer LK, Craske MG, Katz ER (2000). Children’s memories for painful cancer treatment procedures: implications for distress. Child Dev.

[12] Boyce WT, Barr RG, Zeltzer LK (1992). Temperament and the psychobiology of stress. Pediatrics.

[13] Conte PM, Walco GA, Kimura Y (2003). Temperament and stress response in children with juvenile primary fibromyalgia syndrome. Arthritis Rheum.

[14] Rocha EM, Prkachin KM (2006). Temperament and pain reactivity predict health behaviour seven years later. J Pediatr Psychol.

[15] Rocha EM, Prkachin KM, Beaumont SA, Hardy C, Zumbo B (2003). Pain reactivity and somatization in kindergarten-aged children. J Pediatr Psychol.

[16] McCarthy AM, Kleiber C, Hanrahan K, Zimmerman MB, Westhus N, Allen S (2010). Factors explaining Children’s responses to intravenous needle insertions. Nurs Res.

[17] Duff AJA (2003). Incorporating psychological approaches into routine paediatric venepuncture. Arch Dis Child.

[18] Bagnasco A, Pezzi E, Rosa F, Fornoni L, Sasso L (2012). Distraction techniques in children during venipuncture: an Italian experience. J Prev Med Hyg.

[19] Noel M, McMurtry CM, Chambers CT, McGrath PJ (2010). Children’s memory for painful procedures: the relationship of pain intensity, anxiety, and adult behaviors to subsequent recall. J Pediatr Psych.

[20] Du J, Champion GD, Yap C (2008). Theories of fear acquisition: the development of needle phobia in children. Pediatric Pain Letter.

[21] Muris P, Merckelbach H, Vasey MW, Dodds MR (2001). The Etiology of Childhood Specific Phobia: A Multifactorial Model. The Developmental Psychopathology of Anxiety.

[22] Merritt C (2014). Fear and loathing in the ER: managing procedural pain and anxiety in the pediatric emergency department. R I Med J.

[23] Uman LS, Chambers CT, McGrath PJ, Kisely S (2008). A systematic review of randomized controlled trials examining psychological interventions for needle-related procedural pain and distress in children and adolescents: an abbreviated cochrane review. J Pediatr Psychol.

[24] Srouji R, Ratnapalan S, Schneeweiss S (2010). Pain in children: assessment and nonpharmacological management. Int J Pediatr.

[25] Kazak AE, Penati B, Brophy P, Himelstein B (1998). Pharmacologic and psychologic interventions for procedural pain. Pediatrics.

[26] Souders MC, Freeman KG, DePaul D, Levy SE (2002). Caring for children and adolescents with autism who require challenging procedures. Pediatr Nurs.

[27] Spagrud LJ, von Baeyer CL, Ali K, Mpofu C, Fennell LP, Friesen K, Mitchell J (2008). Pain, distress, and adult-child interaction during venipuncture in pediatric oncology: an examination of three types of venous access. J Pain Symptom Manage.

[28] Festini F, Neri S, Crinelli S, Bastiani C, Galici V, de Martino M, Caprilli S (2008). Pain perception related to venipuncture in children with cystic fibrosis, compared to healthy children. Int Nurs Perspect.

[29] Sermet-Gaudelus I, De Villartay P, de Dreuzy P, Clairicia M, Vrielynck S, Canoui P, Kirzsenbaum M, Singh-Mali I, Agrario L, Salort M, Charron B, Dusser D, Lenoir G, Hubert D (2009). Pain in children and adults with cystic fibrosis: a comparative study. J Pain Symptom Manage.

[30] Madge S, Festini F, Neri S, Ballarin S, Anbar R, Annam A, Armoni Y, Boulanger L, Bregnballe V, Crews B, De Vries J, Douthit J, Elworthy S, Erwander I, Ferguson ML, Green R, Hennessey R, Heydendael M, Jokinen L, Kerbrat M, Laraya-Cuasay L, Lomas F, McMullen A, Nation K, Peterson M, Tolomeo T (2006). Procedural pain in childrem with cystic fibrosis: an international survey on the methods used by CF Centers to prevent and reduce it. J Cyst Fibros.

[31] Twycross A (1998). Dispelling modern day myths about children’s pain. Child Health Care.

[32] Van Hulle VC (2005). Nurses’ knowledge, attitudes and practices: regarding children’s pain. MCN Am J Matern Child Nurs.

[33] Zelter LK, Anderson CT, Schechter NL (1990). Pediatric pain: current status and new directions. Curr Probl Pediatr.

[34] Collier J, Pattinson H (1997). Attitudes to children’s pain: exploding the pain myth. Paediatr Nurs.

[35] Rømsing J, Dremstrup Skovgaard C, Friis SM, Henneberg SW (2014). Procedure-related pain in children in a Danish UniversityHospital. A qualitative study. Ped Anesth.

[36] Collins JG (1997). Prevalence of selected chronic conditions: United States, 1990–1992. National Center for health Statistics. Vital Health Stat.

[37] Duff AJ, Gaskell SL, Jacobs K, Houghton JM (2012). Management of distressing procedures in children and young people: time to adhere to the guidelines. Arch Dis Child.

[38] Hockenberry MJ, Wong DL (2004). Wong’s Clinical Manual of Pediatric Nursing.

[39] Wong DL, Baker CM (1988). Pain in children: comparison of assessment scales. Pediatr Nurs.

[40] Elliott CH, Jay SM, Woody P (1987). An observation scale for measuring children’s distress during medical procedures. J Pediatr Psychol.

[41] Goodenough B, Champion GD, Laubreaux L, Tabah L, Kampel L (1998). Needle pain severity in children: does the relationship between self-report and observed behaviour vary as a function of age?. Aust J Psychol.

[42] Fowler-Kerry S, Lander J (1991). Assessment of sex differences in children’s and adolescents’ self-reported pain from venipuncture. J Pediatr Psychol.

